# Application of Genomic Epidemiology of Pathogens to Farmed Yellowtail Fish Mycobacteriosis in Kyushu, Japan

**DOI:** 10.1264/jsme2.ME24011

**Published:** 2024-06-20

**Authors:** Takayuki Wada, Shiomi Yoshida, Takeshi Yamamoto, Lisa Nonaka, Yukari Fukushima, Chie Nakajima, Yasuhiko Suzuki, Masayuki Imajoh

**Affiliations:** 1 Department of Microbiology, Graduate School of Human Life and Ecology, Osaka Metropolitan University, Osaka, Japan; 2 Osaka International Research Center for Infectious Diseases, Osaka Metropolitan University, Osaka, Japan; 3 Clinical Research Center, National Hospital Organization Kinki-chuo Chest Medical Center, Sakai, Osaka, Japan; 4 Azuma-cho Fisheries Cooperative Association, Izumi, Kagoshima, Japan; 5 Faculty of Human Life Sciences, Shokei University, Kumamoto, Kumamoto, Japan; 6 Division of Bioresources, International Institute for Zoonosis Control, Hokkaido University, Sapporo, Hokkaido, Japan; 7 Division of Research Support, Institute for Vaccine Research and Development, Hokkaido University, Sapporo, Hokkaido, Japan; 8 Laboratory of Fish Disease, Aquaculture Course, Department of Marine Resource Science, Faculty of Agriculture and Marine Science, Kochi University, Nankoku, Kochi, Japan

**Keywords:** *Mycobacterium pseudoshottsii*, fish mycobacteriosis, public health, fish farm

## Abstract

To investigate mycobacterial cases of farmed yellowtail fish in coastal areas of western Japan (Kagoshima, Kyushu), where aquaculture fisheries are active, *Mycobacterium pseudoshottsii*, the causative agent, was isolated from six neighboring fishing ports in 2012 and 2013. A phylogenetic ana­lysis revealed that the strains isolated from one fishing port were closely related to those isolated from other regions of Japan, suggesting the nationwide spread of a single strain. However, strains from Japan were phylogenetically distinct from those from the Mediterranean and the United States; therefore, worldwide transmission was not observed based on the limited data obtained on the strains exami­ned in this study. The present results demonstrate that a bacterial genomic ana­lysis of infected cases, a mole­cular epidemiology strategy for public health, provides useful data for estimating the prevalence and transmission pathways of *M. pseudoshottsii* in farmed fish. A bacterial genome ana­lysis of strains, such as that performed herein, may play an important role in monitoring the prevalence of this pathogen in fish farms and possible epidemics in the future as a result of international traffic, logistics, and trade in fisheries.

*Mycobacterium marinum* is a well-known contributor to fish mycobacteriosis (Gauthier and Rhodes, 2009; Aubry *et al.*, 2017; Hashish *et al.*, 2018), which causes disease in both freshwater and saltwater fish, whether they are farmed or wild (Jacobs *et al.*, 2009; Gauthier and Rhodes, 2009). Humans may also be infected with this bacterium when they are exposed to contaminated aquatic environments or marine animals (Decostere *et al.*, 2004; Hashish *et al.*, 2018). Detailed genetic sequencing revealed the extensive genetic diversity of *M. marinum*, which has many fish disease-causing subspecies, including *M. liflandii* (*M. ulcerans* ecovar *liflandii*), *M. shottsii*, and *M. pseudoshottsii* (Yip *et al.*, 2007; Tobias *et al.*, 2013; Hikima *et al.*, 2016; Das *et al.*, 2018; Gauthier *et al.*, 2022). *M. ulcerans*, which causes Buruli ulcer (Guarner, 2018), a severe invasive skin disease in humans, is closely related to these species (Stinear *et al.*, 2007; Yip *et al.*, 2007). These species share more than 98% genome sequence identity and, thus, are collectively referred to as the *M. marinum* complex (Stinear *et al.*, 2007; Tobias *et al.*, 2013; Hikima *et al.*, 2016; Das *et al.*, 2018; Gauthier *et al.*, 2022).

*M. pseudoshottsii* (*Mps*) was initially detected during a mycobacteriosis epizootic in 2007 (Rhodes *et al.*, 2005). Since then, the organism’s geographical distribution range and the various fish species exposed to it have been increasingly reported. In 2009, this species was also isolated from striped bass in the New York Bight (Stine *et al.*, 2009). Furthermore, cases of infection with this organism in aquaculture farms have been reported in Japan (Nakanaga *et al.*, 2012), with accompanying descriptions of mass mortality. Another report was documented in 2020, describing cases of infected farmed fish in Italy (Mugetti *et al.*, 2020). In 2022, the organism was detected in sardines held in aquariums in Japan (Komine *et al.*, 2022). In 2020–2021, this organism caused many cases of infection in an aquaculture facility in Israel raising imported food fish from the United States (Davidovich *et al.*, 2023). Consequently, there is apprehension that this pathogen may be spreading surreptitiously not only in aquaculture fisheries, but also among fish bred in different facilities worldwide, based on reports of sporadic cases of its occurrence from diverse sources.

The identification of possible sources for the transmission of *Mps* is critical for mitigating the damage caused by the spread of this infection. Examinations of genetic variations among strains allow for the estimation of their origins. An Italian study analyzed the partial sequences of *hsp65*, one of the housekeeping genes, from several strains isolated from three different aquaculture farms (Mugetti *et al.*, 2020). Although these strains were classified into only three groups based on their sequences, the findings obtained indicated different sources of infection. More detailed techniques using high-throughput sequencers have recently been effectively employed to identify the source of not only *M. tuberculosis* cases in humans (Walker *et al.*, 2013a, 2013b) but also *M. bovis* in wild animals (Biek *et al.*, 2012). This method is known as a mole­cular epidemiological ana­lysis and has been widely used for the majority of pathogens (Grad and Lipsitch, 2014; Kao *et al.*, 2014), including in the COVID-19 pandemic (Rockett *et al.*, 2020), as well as in mycobacteriosis.

To estimate the transmission routes of pathogens for a mole­cular epidemiological ana­lysis, it is critical to sequence the full-length genomes of each strain and strictly elucidate their identity based on single-nucleotide variations (SNVs). In the case of *Mps*, four full-length genome sequences (the type strain L15, synonym JCM 15466^T^ and DSM 45108, originally isolated from a striped bass in Chesapeake Bay; AR, isolated from *Argyrosomus regius* in Western Greece; NJB1907-Z4, isolated from an aquarium-reared Japanese sardine in Japan; YM-3, isolated from cultured yellowtail fish in Japan) are currently available in the NCBI and serve as reference sequences (Imajoh *et al.*, 2023). By selecting an appropriate reference from these sequences, nucleotide variations among strains may be successfully identified, which may enable us to estimate factors of infection and/or the background of transmission.

A genome ana­lysis of this bacterium has provided only a partial understanding of phylogenetic relationships and a detailed genome ana­lysis has not yet been conducted. In the present study, we report the isolation of the causative organisms from an outbreak of acid-fast bacilli in cultured yellowtail fish in 2012 in Japan, demonstrating that these were cases of *Mps* infection, and elucidated their genome sequences. In addition, we verified genetic relatedness and geographical relationships among the strains, which serves as a mole­cular epidemiological approach to the setting.

## Materials and Methods

### Strains

The 12 strains included in the present study were isolated from fish carcasses suspected to have died due to mycobacterial‍ ‍in­fection. These carcasses were brought to Azuma-cho Fisheries Cooperative Association from six ports in the western area of Kagoshima Prefecture in 2012 and 2013. Yellow colonies of mycobacterial strains were obtained by culturing lesion samples at 25°C for 2‍ ‍weeks on Ogawa’s media (Kyokuto Pharmaceutical Industrial).

To accurately identify the mycobacterial species, bacterial strains were sent to the Clinical Research Center of the National Hospital Organization Kinki-chuo Chest Medical Center. Photochromogenicity was confirmed based on Runyon’s classification. Four partial genes (16S rRNA, ITS, *hsp65*, and *rpoB*), which have been used to identify mycobacterial species (Roth *et al.*, 1998; Kim *et al.*, 2006), were sequenced by Eurofin Japan.

### Genome sequencing

Genomic DNA samples were purified from cultured bacteria as previously described (Belisle and Sonnenberg, 1998) to prepare libraries for short-read sequencing using the QIAseq FX library preparation kit (QIAGEN). MiSeq (Illumina) was used for short-read sequencing with MiSeq Sequencing Kit v3 (600-cyc) (Illumina). Raw read data were deposited in the DDBJ Sequence Read Archive (DRA) under the accession numbers DRR506146-DRR506157.

### Genome ana­lysis with *M. marinum* complex strains

After filtering low-quality or contamination reads using the ‘quality trimming’ and ‘removal of mapped reads’ functions in the CLC Genomics Workbench v20.0.4 (QIAGEN), the sequence reads of each strain were assembled using SPAdes genome assembler v3.15.4 (Prjibelski *et al.*, 2020). The contigs obtained were used for a core-genome ana­lysis in combination with 19 complete genome sequences of the *M. marinum* complex registered in the NCBI database (Table S1). Short-read sequences obtained from three *Mps* strains reported from Israel (Davidovich *et al.*, 2023) were also downloaded from the database, assembled in the same manner as described above, and integrated into this ana­lysis. The core SNVs were retrieved using kSNP4.0, an upgraded version of kSNP3 (Gardner *et al.*, 2015). The k-mer was 19 and covered >99% of the genome sequences using Kchooser4, a program bundled with kSNP4.0. The fraction of core k-mers (FCK) was also checked using this program. The matrix of SNV data was used as an alignment to construct maximum likelihood trees using IQ-TREE ver. 1.6.12 (Nguyen *et al.*, 2015). The substitution model was selected using the option “-m MFP+ASC” in the command line, which allowed for model selection under the Bayesian information criterion with ascertainment bias correction. The tree files constructed using this tool were visualized using iTOL ver. 6.7.6 (Letunic and Bork, 2021).

### Detection of SNVs with a close reference

Based on the core genome phylogenetic tree, YM-3 was selected as the reference strain for further ana­lyses. The short reads of the 12 *Mps* strains and NJB1907-Z4 (accession no. DRR337915) were mapped to the complete genome sequence of YM-3 (Imajoh *et al.*, 2023) using the CLC Genomics Workbench v20.0.4 (QIAGEN) to detect SNVs. The SNVs were called only from reference regions with a mapping coverage >10 and not containing non-specific mapping reads.

After the filtration of heterogeneous SNVs (<80% variant calls), the alleles of the SNVs were compared between the 12 strains and the reference YM-3. All nucleotides corresponding to the SNVs were concatenated to each nucleotide sequence for each strain and used to construct a median-joining tree (Bandelt *et al.*, 1999) with POPART ver. 1.7 (Leigh and Bryant, 2015). All SNV alleles of JCM 15466 were also confirmed by comparisons with the YM-3 genome sequence using the Nucmer program in Mummer3 (Kurtz *et al.*, 2004).

## Results

### Occurrence and symptoms of *Mps* infection

**Fig. 1.**
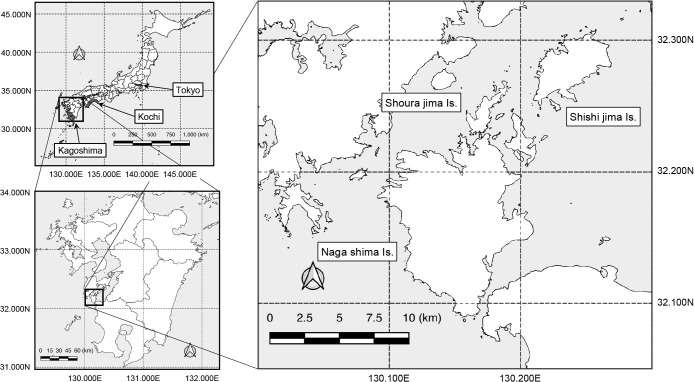
Geographic setting of the present study. Mycobacteriosis cases in cultured yellowtail were collected from six ports at three islands (Shishijima Is., Shourajima Is., and Nagashima Is.) of the Amakusa Archipelago, located in the western coast of Kagoshima Prefecture, Japan. This area is located approximately 1,000‍ ‍km west of Tokyo and is well known for its fish-farming industry. Kochi Prefecture, from which reference strain YM-3 was isolated, is also indicated. Map data were retrieved from the Database of Global Administrative Areas (GADM) and digital national land information provided by the Ministry of Land, Infrastructure, Transport, and Tourism. QGIS 3.30 was used to create the map.

A part of the Amakusa archipelago in the western area of Kagoshima Prefecture, located in southern Kyushu, Japan (Fig. 1), is known for its active food fish aquaculture, particularly for yellowtail and red snapper. During yellowtail farming in these areas, primarily during summer (July to September), fatalities and a high number of sick individuals among cultured fish have been observed (up to ~5% of the total number of fish in a fish tank), particularly in zero-year-old fish, due to infection with acid-fast bacilli. White granulomas are commonly found on the gills and internal organs, primarily the liver and kidneys. Ulcerative lesions are occasionally observed on the skin surface, which are diagnosed as mycobacterial infections through the acid-fast staining of tissue sections.

All yellowtail fish carcasses collected for examination (377 cases from 2012 and 337 from 2013) from six ports were found to be anally open with no skin lesions. Autopsy revealed the presence of jaundice and kidney nodules in 26 cases from 2012 and 53 from 2013, which strongly indicated mycobacterial infection. Twelve slow-growing acid-fast bacilli were isolated from these carcasses (Table 1), all of which were classified as Runyon I, including *M. marinum*, based on their photochromogenicity (data not shown). These strains were culturable at room temperature (approximately 25°C), but did not grow at 37°C. Of the 12‍ ‍strains, seven were isolated from samples provided by Port A on Shishijima Island, where mycobacteriosis cases were monitored for two years. The remaining five strains were selected from different fishing ports (Ports B to F) on Moroshojima Island and Nagashima Island, where cases were monitored in 2012 only. To anonymize each port, only the approximate distances between ports are shown (Table 2).

The partial nucleotide sequences of four genes (16S rRNA, ITS, *hsp65*, and *rpoB*), which are essential housekeeping genes, were also identified in these strains. The results obtained showed that all these strains belonged to the *M. marinum* complex (data not shown). Notably, all 12 strains showed c.637C>T in *hsp65*, which is a characteristic feature of *Mps* (Nakanaga *et al.*, 2012).

### Core genome comparison with the *M. marinum* complex

**Fig. 2.**
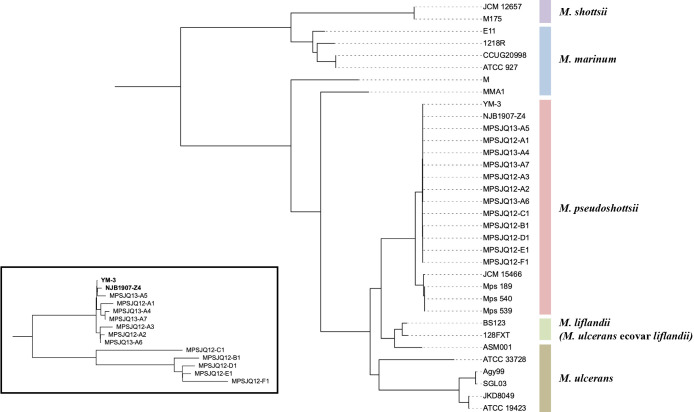
A phylogenetic tree constructed with core single-nucleotide variations (SNVs) (65,625 bp) of the *Mycobacterium marinum* complex and 12 *Mycobacterium pseudoshottsii* (*Mps*) strains isolated in this study. SNVs were detected on the core k-mers (k=19), corresponding to ~77% of the sequence length of the bacterial genome. The assembled contigs of three *Mps* strains isolated from the West Mediterranean Sea (designated as *Mps*189, *Mps*539, and *Mps*540) were also included. The registered genome sequences were retrieved from the NCBI database (Genbank IDs are listed in Table S1). The HKY model with base frequency counted directly from the alignment (HKY+F) was used as the substitution model. An enlarged tree of the core SNVs (65,974 bp) of the 14 *Mps* strains isolated in Japan is shown in the inset. SNVs were also detected on the core k-mers (k=19), corresponding to ~99% of the bacterial genome sequence length. The transversion model with base frequency counted directly from the alignment under ascertainment bias correction (TVM+F+ASC) was used as the substitution model.

All bacterial strains were genomically sequenced, and contigs of approximately 6.0‍ ‍Mb were assembled. These contig sequences were incorporated into the genome sequences of *M. marinum* complex strains available from the nucleotide database for a core genome ana­lysis, along with the contig sequences of three *Mps* strains (named as iso­lates 189, 539, and 540) isolated from the East Mediterranean Sea of Israel (Davidovich *et al.*, 2023). The core genome phylogenetic tree showed that the sequences of the two registered *Mps* strains (YM-3 and NJB1907-Z4) and those of the 12 strains in the present study were almost identical, whereas those of three Mediterranean strains were similar to that of the reference strain JCM 15466 (Fig. 2). An enlargement of the tree of these 14 strains showed that strains isolated from Port A were very closely related to the two well-known strains, while strains isolated from other ports belonged to another branch (Fig. 2, inset).

### Comparison based on single-locus variants (SLVs)

**Fig. 3.**
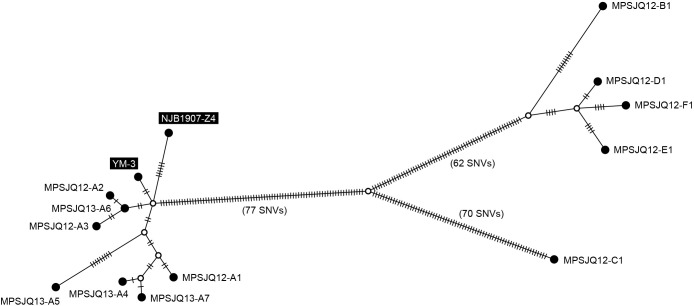
A linkage tree constructed using 264 positions of SNVs of *Mps* strains identified through a mapping ana­lysis to a complete reference genome (YM-3: AP028092.1). Closed circles represent each strain, with the strain name indicated near them. The names of two strains isolated in other studies are shown in inverted black and white labels. Open circles indicate branching points on the tree; however, no strains corresponding to these branching points were found in this study. The number of hatch marks on each branch corresponds to the number of SNVs. The number of SNVs corresponding to the three longest branches recognized in the tree was indicated numerically in addition to the hatches for clarity.

The 12 *Mps* strains obtained in the present study were very closely related to the strains isolated in Japan (YM-3 and NJB1907-Z4, isolated in 1986 and 2019, respectively), which prompted us to integrate them for a detailed genetic linkage ana­lysis. A median-joining tree was constructed based on all SNVs (264 positions, listed in Table S2) detected through a mapping ana­lysis of the 13 strains, using the YM-3 complete genome sequence as a reference (Fig. 3). These strains were divided into two major subgroups and a separated unique strain (MPSJQ12-C1; isolated from Port C). All strains from Port A were highly similar to the two strains (YM-3 and NJB1907-Z4) isolated from a separate area of Japan, implying the recent transmission of these strains across a wide area of the country. In contrast, the four isolates from the respective ports (B, D, E, and F) were also closely related to each other, indicating local transmission, in contrast to Port A. Although these four ports are located on two geographically close islands, MPSJQ12-C1 was unique from the other strains because it had a higher number of SNVs even though it originated from Port C, which is located on the same island as Ports B and D.

## Discussion

This is the first study to introduce a mole­cular epidemiological approach to investigate the source of fish disease caused by *Mps*, a closely related species of *M. marinum*, which is etiological in aquatic animals. Phylogenetically, this species is distinct from *M. ulcerans* and its ecovar *liflandii*, which are also derived subspecies (Fig. 2). In contrast to these two subspecies, the number of cases of infection owing to *Mps* has been increasing in industrial fisheries, and there are concerns that its economic impact may be severe (Stine *et al.*, 2009; Mugetti *et al.*, 2020; Davidovich *et al.*, 2023). This situation led us to anticipate that controlling infection by this species in fisheries will be required in the future. In the present study, strains from only 12 cases were analyzed, out of the many cases that would have occurred within the nearshore area of Kagoshima Prefecture, Japan over a 2-year period, of which one port was involved in the collection of strains over a 2-year period. Mugetti *et al.* (2020) also collected strains in a similar setting, and a more detailed ana­lysis with additional strains will provide a more accurate estimation of transmission pathways. The introduction of a genomic epidemiological ana­lysis, which is used in human public health, to these cases may lead to the more effective management of farmed fish and the possibility of mitigating or preventing economic damage caused by infectious disease.

The comparative genomic ana­lysis of the strains in this study allowed us to arrive at the following three inferences. The strains isolated from cases that occurred in Port A were highly similar to strains previously isolated from other distant regions of Japan (Fig. 1: YM-3 from Kochi and NJB1907-Z4 from Tokyo), suggesting that nationwide transmission routes exist. Furthermore, local transmission in several ports (B, D, E, and F) may be inferred from the similarities of strains from the four ports, which were distinct from those isolated from Port A. Moreover, the strain isolated from Port C was unique, suggesting that the source of infection differed from that of the other ports or that it was port-specific. Genomic data on strains from cases within Port A analyzed in this study indicate nationwide spread, as‍ ‍mentioned earlier; however, no correlation was found between reports from other regions, indicating uncertainty regarding the source of the infection. Yellowtails are sometimes subject to “intermediate rearing”, in which young fish are reared for a year and then transferred to other ports, which may be a possible route of transmission owing to traffic among ports. On the other hand, the group of strains was isolated from four different ports (B, D, E, and F), suggested that a genome-based study will provide a regional basis for the spread of this pathogen.

It is important to note that transmission routes among these clonal strains may be overestimated because the mutation rate of this species may be slow, similar to that in other mycobacteria (*e.g.*, in *M. tuberculosis*, <0.5 SNP/year) (Walker *et al.*, 2013a; Guerra-Assunção *et al.*, 2015). Therefore, phylogenetic clusters comprising strains from Port A and from the references may be interpreted as originating from unrelated subgroups that had branched independently from a common ancestor. However, even under such an assumption, it may be argued that the evidence obtained also supports nationwide spread. However, more strains need to be collected from each port for multiple cases for a comparative genomic ana­lysis.

The worldwide *Mps* epidemic, which may occur because of trade and logistics, may be monitored through genome comparisons of strains accumulated internationally from cases of *Mps* infection. *Mps* strains from Mediterranean farmed fish in Italy and Israel were genome-phylogenetically distinct from Japanese strains (Fig. 2). The strains from Israel were phylogenetically close to the type strain JCM 15466^T^ isolated in the United States; therefore, the possibility of international fish trade being responsible for the spread of infection has been noted (Mugetti *et al.*, 2020). The strains from Japan analyzed in the present study were distinct from these strains, indicating that the dissemination of these strains may be limited to Japan, whereas cross-transmission among Western countries has not yet been exami­ned. The future distribution of these distinct strains may lead to a more global mixture and genome-based monitoring may be able to accurately characterize the outbreak. Since the cases we analyzed occurred in 2012, constant monitoring may be required for prompt implementation.

In summary, the present study demonstrated that strain comparisons based on a genomic ana­lysis of *Mps* outbreaks in breeding fish may provide valuable insights for epidemic monitoring. The results obtained also indicate that the accumulation of detailed genomic data on a pathogen may generate a public health contribution to the search for the origin of mycobacterial diseases in aquaculture fisheries.

## Citation

Wada, T., Yoshida, S., Yamamoto, T., Nonaka, L., Fukushima, Y., Nakajima, C., et al. (2024) Application of Genomic Epidemiology of Pathogens to Farmed Yellowtail Fish Mycobacteriosis in Kyushu, Japan. *Microbes Environ ***39**: ME24011.

https://doi.org/10.1264/jsme2.ME24011

## Supplementary Material

Supplementary Material

## Figures and Tables

**Table 1.** Clinical strains of *Mycobacterium pseudoshottsii* isolated from yellowtail fish in aquaculture facilities of Japan

Strain	Date of Isolation	Isolation port	Age
MPSJQ12-A1	Sep. 5, 2012	A	zero-years-old
MPSJQ12-A2	Sep. 6, 2012	A	zero-years-old
MPSJQ12-A3	Oct. 15, 2012	A	one-year-old
MPSJQ13-A4	Aug. 28, 2013	A	zero-years-old
MPSJQ13-A5	Sep. 13, 2013	A	one-year-old
MPSJQ13-A6	Sep. 30, 2013	A	one-year-old
MPSJQ13-A7	Nov. 8, 2013	A	zero-years-old
MPSJQ12-B1	Aug. 21, 2012	B	zero-years-old
MPSJQ12-C1	Oct. 15, 2012	C	zero-years-old
MPSJQ12-D1	Sep. 13, 2012	D	zero-years-old
MPSJQ12-E1	Aug. 29, 2012	E	zero-years-old
MPSJQ12-F1	Sep. 14, 2012	F	one-year-old

**Table 2.** Geographic relationship of fishing ports from which mycobacteriosis cases were analyzed in the present study

Port	Island	Approx. distance to another port (km)					
		Port A	Port B	Port C	Port D	Port E	Port F
A	Shishijima Is.	0.0	3.8	5.2	6.2	6.2	8.0
B	Shourajima Is.	3.8	0.0	2.0	2.7	3.7	5.2
C	Shourajima Is.	5.2	2.0	0.0	1.1	1.8	3.2
D	Shourajima Is.	6.2	2.7	1.1	0.0	2.2	2.8
E	Nagashima Is.	6.2	3.7	1.8	2.0	0.0	1.7
F	Nagashima Is.	8.0	5.2	3.2	2.8	1.7	0.0
