# Supplementary material for: Application of Genomic Epidemiology of Pathogens to Farmed Yellowtail Fish Mycobacteriosis in Kyushu, Japan
Source: Microbes Environ. 2024 Jun 20;39(2):ME24011. doi: 10.1264/jsme2.ME24011 (PMC11220446; doi:10.1264/jsme2.ME24011)
Supplement: Supplementary file 1 — Supplementary Material [file 39_24011_s1.pdf]

Table S1 Genome sequences of *Mycobacterium marinum* complex strains used for this study

Strain	Species	Accession ID
JCM 12657	*M. shottsii*	AP022572.1
M175	*M. shottsii*	CP014860.2
E11	*M. marinum*	HG917972.2
1218R	*M. marinum*	CP025779.1
CCUG2098	*M. marinum*	CP024190.1
ATCC 927	*M. marinum*	AP018496.1
M	*M. marinum*	CP000854.1
MMA1	*M. marinum*	CP058277.1
YM-3	*M. pseudoshottsii*	AP028092.1
NJB1907-Z4	*M. pseudoshottsii*	AP026367.1
JCM 15466	*M. pseudoshottsii*	AP018410.1
ASM001	*M. liflandi*	CP023138.1
128FXT	*M. liflandi*	CP003899.1
BS123	*M. ulcerans*	CP083741.1
ATCC 33728	*M. ulcerans*	AP017624.1
Agy99	*M. ulcerans*	CP000325.1
SGL03	*M. ulcerans*	LR135168.1
JKD8049	*M. ulcerans*	CP085200.1
ATCC 19423	*M. ulcerans*	CP092429.1

Table S2 Single nucleotide variations (SLVs) identified in 13 strains of *Mycobacterium pseudoshottsii* to the genome sequence of YM-3 (AP028092.1)

Position No.	Reference	Variation	Strains													Locus tag	Gene	Amino acid change
			NJB1907-Z4	MPSJQ12-A1	MPSJQ12-A2	MPSJQ12-A3	MPSJQ13-A4	MPSJQ13-A5	MPSJQ13-A6	MPSJQ13-A7	MPSJQ12-B1	MPSJQ12-C1	MPSJQ12-D1	MPSJQ12-E1	MPSJQ12-F1			
33379	C	T									x		x	x	x	YM3MPS_00300		synonymous
36475	T	C									x	x	x	x	x	YM3MPS_00320		synonymous
47628	T	C									x	x	x	x	x	YM3MPS_00480		synonymous
59339	T	C										x				YM3MPS_00580		Gln12Arg
100058	T	C									x		x	x	x	intergenic	-	-
109109	C	T										x				YM3MPS_00960		synonymous
156395	C	A	x													YM3MPS_01460		Gly342Val
195192	G	T	x	x	x	x	x	x	x	x	x	x	x	x	x	YM3MPS_01820		Gly351Cys
197346	C	T											x	x	x	YM3MPS_01830		synonymous
197440	G	C									x	x	x	x	x	YM3MPS_01830		Gly459Arg
212024	T	C						x								YM3MPS_01960		Thr84Ala
227035	G	A									x		x	x	x	YM3MPS_02100		Glu865Lys
275046	C	T			x	x			x							YM3MPS_02510	*katA*	Arg90Cys
290864	A	C					x			x						YM3MPS_02650		Thr74Pro
294743	C	T									x		x	x	x	YM3MPS_02680		Gly112Asp
310254	T	C									x	x	x	x	x	YM3MPS_02810		synonymous
319094	T	C									x		x	x	x	YM3MPS_02920	*cyp* 137	synonymous
330895	A	G									x	x	x	x	x	YM3MPS_03050	*nth*	Arg7Gly
411227	A	G										x				YM3MPS_03840		Asn323Ser
438078	C	T									x		x	x	x	YM3MPS_04090		synonymous
472044	G	A						x								YM3MPS_04450		synonymous
507947	A	G									x	x	x	x	x	YM3MPS_04690	*fadE2*	synonymous
513401	A	G				x										YM3MPS_04740	*pntB*	Lys472Glu
524619	A	G									x	x	x	x	x	YM3MPS_04860		synonymous
535778	C	G									x	x	x	x	x	YM3MPS_04990		synonymous
571116	G	A									x		x	x	x	YM3MPS_05320		Pro977Leu
571647	G	A									x	x	x	x	x	YM3MPS_05320		Ala800Val
576090	C	A									x	x	x	x	x	intergenic	-	-
578981	T	G									x	x	x	x	x	intergenic	-	-
580103	G	A									x		x	x	x	YM3MPS_05380		Gly141Arg
629918	C	T										x				YM3MPS_05780		synonymous
635352	T	C									x	x	x	x	x	YM3MPS_05820		Asp406Gly
675339	C	T										x				YM3MPS_06170		Leu32Phe
686266	T	C										x				YM3MPS_06290		Asp132Gly
706228	G	A		x			x	x		x						YM3MPS_06480		synonymous
707297	A	G										x				intergenic	-	-
711060	C	T									x		x	x	x	YM3MPS_06520		synonymous
725912	G	A										x				YM3MPS_06630		Arg175Trp
727213	C	T									x	x	x	x	x	YM3MPS_06640	*tam*	Ala208Val
743426	G	A										x				YM3MPS_06820		Val159Met
756730	A	G									x	x	x	x	x	YM3MPS_06930		Asp34Gly
763068	C	T						x								YM3MPS_06990		Arg380Cys
786258	C	T	x													YM3MPS_07160		His71Tyr
798103	C	T										x				YM3MPS_07260		synonymous
859471	G	A									x		x	x	x	intergenic	-	-
863320	T	C									x	x	x	x	x	YM3MPS_07760		synonymous
889017	G	C									x	x	x	x	x	YM3MPS_08010		Asp6Glu
896594	G	C										x				YM3MPS_08090		Asp357Glu
896697	G	C									x	x	x	x	x	YM3MPS_08090		Thr323Ser
897837	C	T									x		x	x	x	intergenic	-	-
914323	C	T									x		x	x	x	YM3MPS_08210	*thiE*	synonymous
919734	G	A												x		YM3MPS_08290	*lpqL*	Gly79Arg
950320	A	G		x			x			x						YM3MPS_08550	*sodC*	synonymous
980962	C	T									x		x	x	x	intergenic	-	-
997240	C	T									x		x	x	x	YM3MPS_08960	*lpdC*	synonymous
1010193	C	G										x				intergenic	-	-
1020211	T	C									x	x	x	x	x	YM3MPS_09170		synonymous
1028408	A	G											x			YM3MPS_09250	*senX3*	Asp146Gly
1036928	G	T										x				YM3MPS_09330		synonymous
1050529	A	G										x				YM3MPS_09470		Met69Thr
1083697	C	T									x	x	x	x	x	YM3MPS_09750		synonymous

1121542	T	C									x	x	x	x	x	YM3MPS_10140		synonymous
1162282	C	T									x	x	x	x	x	intergenic	-	-
1182359	A	G									x					YM3MPS_10700		Asp563Gly
1193165	T	C									x	x	x	x	x	YM3MPS_10750	*recB*	Ile164Val
1244998	G	A										x				YM3MPS_11180		synonymous
1328326	T	C									x		x	x	x	YM3MPS_12130	*infA*	synonymous
1336883	A	G									x		x	x	x	YM3MPS_12230	*PPE25*_1	Val44Ala
1337520	T	C										x				YM3MPS_12240		synonymous
1340283	T	C									x	x	x	x	x	YM3MPS_12260		Asp74Gly
1345587	T	C									x	x	x	x	x	YM3MPS_12300		synonymous
1394842	T	C									x		x	x	x	YM3MPS_12750		Ser215Gly
1397923	C	T									x		x	x	x	YM3MPS_12790		Gly370Ser
1422345	T	C									x	x	x	x	x	YM3MPS_12980		Leu813Ser
1498536	C	T	x													intergenic	-	-
1504612	C	T										x				YM3MPS_13690	*punA*	synonymous
1568110	C	T									x		x	x	x	YM3MPS_14180		synonymous
1572669	A	G									x	x	x	x	x	YM3MPS_14220		synonymous
1578049	A	C									x		x	x	x	YM3MPS_14270		Val212Gly
1612759	C	T									x		x	x	x	intergenic	-	-
1628375	C	T						x								YM3MPS_14720		synonymous
1634595	T	C				x										YM3MPS_14780		synonymous
1665493	C	T									x	x	x	x	x	YM3MPS_15110		synonymous
1667980	T	C									x	x	x	x	x	YM3MPS_15130	*moeZ*	Val145Ala
1690260	C	T									x		x	x	x	YM3MPS_15310		synonymous
1722377	G	A									x	x	x	x	x	intergenic	-	-
1732536	A	G												x		YM3MPS_15690	*nuoN*	Leu156Pro
1784253	C	T									x	x	x	x	x	YM3MPS_16190	*mrp*	Gly362Ser
1784863	A	G									x		x	x	x	YM3MPS_16190	*mrp*	synonymous
1789008	G	A										x				YM3MPS_16250		Ala359Val
1829408	C	A									x		x	x	x	YM3MPS_16560		synonymous
1831288	T	C										x				YM3MPS_16590		Asp131Gly
1837562	A	T										x				YM3MPS_16660		Thr112Ser
1865243	A	G									x	x	x	x	x	YM3MPS_16940		Arg424Gly
1873843	G	A									x	x	x	x	x	YM3MPS_17010		Gly355Asp
1881471	T	C									x	x	x	x	x	YM3MPS_17050		His160Arg
1946975	A	T										x				YM3MPS_17660		synonymous
1990883	G	A										x				YM3MPS_18040	*mpt53*	Ala83Thr
2020044	A	G									x	x	x	x	x	YM3MPS_18260		Phe174Leu
2065961	G	A										x				YM3MPS_18630		synonymous
2127867	G	A									x					YM3MPS_19250		Ala241Thr
2152966	T	C										x				YM3MPS_19490		Val263Ala
2164062	G	A										x				YM3MPS_19600		Gly292Ser
2255189	C	T													x	YM3MPS_20550		synonymous
2282821	A	C									x		x	x	x	YM3MPS_20830	*tesB2*	Lys281Thr
2290008	C	T										x				YM3MPS_20890		Gly106Ser
2312278	C	T									x		x	x	x	YM3MPS_21090	*fadD9*_1	Asp720Asn
2401515	G	A									x					YM3MPS_21890		Pro128Leu
2448234	G	C									x	x	x	x	x	intergenic	-	-
2519471	A	G					x			x						YM3MPS_22920		Trp239Arg
2532000	T	C											x	x	x	YM3MPS_23030		Val262Ala
2545569	A	G									x		x	x	x	intergenic	-	-
2583118	A	G										x				YM3MPS_23460		Cys8Arg
2595427	T	C									x	x	x	x	x	YM3MPS_23570	*dnaE1*	synonymous
2605482	C	T									x		x	x	x	YM3MPS_23670	*treY*	synonymous
2613008	T	C									x	x	x	x	x	intergenic	-	-
2628929	C	G	x													YM3MPS_23870	*hisB*	Pro130Arg
2637505	C	G										x				YM3MPS_23960	*cphA*	Gly508Ala
2717961	C	T									x	x	x	x	x	YM3MPS_24600	*argB*	synonymous
2737637	A	G											x			YM3MPS_24710	*pks9*	Asp56Gly
2847302	C	G									x	x	x	x	x	YM3MPS_25730		Arg382Pro
2875098	T	G											x	x	x	intergenic	-	-
2889066	G	A									x					YM3MPS_26050		synonymous
2889888	T	G										x				YM3MPS_26060		Met107Leu
2932797	C	T									x		x	x	x	YM3MPS_26470		synonymous
2953918	T	G									x					intergenic	-	-
2969759	G	A									x	x	x	x	x	YM3MPS_26810	*ppe32*_1	Gly20Glu
2994149	G	A										x				YM3MPS_27020	*garA*	Ala132Thr
3027015	C	G									x	x	x	x	x	YM3MPS_27310		synonymous
3033133	G	A									x	x	x	x	x	intergenic	-	-

3035924	A	G									x				YM3MPS_27400	*xfp*	Leu206Pro
3044757	C	A								x		x	x	x	YM3MPS_27480		Gly76Val
3090332	A	G									x				YM3MPS_27960		Cys433Arg
3108291	G	A								x		x	x	x	YM3MPS_28180		Met166Ile
3126087	T	G									x				YM3MPS_28340	*lipD*	Leu322Arg
3144607	G	A									x				YM3MPS_28490		Gly418Asp
3196611	A	G								x	x	x	x	x	intergenic	-	-
3203461	T	C								x	x	x	x	x	YM3MPS_29100		synonymous
3241975	G	T									x				YM3MPS_29520		Ala111Ser
3281116	G	A								x		x	x	x	YM3MPS_29820	*nadR*	Gly112Glu
3305427	G	A								x		x	x	x	YM3MPS_30040		Ala254Thr
3312777	T	C									x				YM3MPS_30130	*fdxA*	Thr32Ala
3313682	G	C								x		x	x	x	YM3MPS_30140		Gln55Glu
3315104	G	A									x				YM3MPS_30170		Arg292Cys
3331349	T	C	x												YM3MPS_30340		Asp105Gly
3342760	G	T									x				YM3MPS_30440		Leu117Met
3449256	G	A								x	x	x	x	x	intergenic	-	-
3484005	G	A								x		x	x	x	YM3MPS_31590		Pro116Ser
3485087	T	C									x				intergenic	-	-
3489539	C	A									x				YM3MPS_31670		Gly9Cys
3587295	G	A								x		x	x	x	intergenic	-	-
3613659	G	A									x				YM3MPS_32810	*ctaD* _1	Thr308Ile
3704682	C	A								x		x	x	x	YM3MPS_33680	*fadD11* _2	synonymous
3710574	C	T								x	x	x	x	x	YM3MPS_33730		Ala349Val
3728773	C	G					x								YM3MPS_33880		synonymous
3734473	C	T									x				YM3MPS_33910	*nifJ* -1	Thr895Ile
3753353	G	A									x				YM3MPS_34100	*fprA* _1	Gly267Arg
3755835	C	T								x	x	x	x	x	YM3MPS_34130		Arg237His
3756593	T	G									x				intergenic	-	-
3764521	A	G								x	x	x	x	x	YM3MPS_34200		synonymous
3766164	G	A								x		x	x	x	YM3MPS_34220		Asp187Asn
3775957	C	A							x						intergenic	-	-
3811498	T	C		x											YM3MPS_34690	*pfkB*	Thr271Ala
3881526	A	G								x	x	x	x	x	YM3MPS_35370		Val2Ala
3890982	C	G								x	x	x	x	x	YM3MPS_35430	*glnN*	synonymous
3939036	G	A								x					YM3MPS_35850		Pro178Leu
3946487	G	T								x	x	x	x	x	YM3MPS_35920		synonymous
4004567	G	A									x				YM3MPS_36360	*plcA*	synonymous
4041508	A	G								x	x	x	x	x	YM3MPS_36640		Tyr248Cys
4044502	G	A									x				YM3MPS_36660		Val556Ile
4099860	G	C								x		x	x	x	YM3MPS_37150		synonymous
4124982	T	G									x				YM3MPS_37350		Val532Gly
4133517	G	A	x			x	x		x						YM3MPS_37420		Gly162Asp
4135721	G	A									x				YM3MPS_37430		Ala132Val
4201726	A	G								x	x	x	x	x	YM3MPS_37920	*plsC*	Val193Ala
4214493	A	G								x	x	x	x	x	YM3MPS_38030	*pdhB*	Cys220Arg
4233288	G	A									x				YM3MPS_38240	*orn*	synonymous
4235570	A	G								x	x	x	x	x	intergenic	-	-
4241692	G	A									x				YM3MPS_38320		Gly244Asp
4242834	C	T								x	x	x	x	x	YM3MPS_38330		Thr253Ile
4248683	G	A									x				YM3MPS_38410		Val167Ile
4279468	T	G								x	x	x	x	x	intergenic	-	-
4286841	T	C								x		x	x	x	intergenic	-	-
4290874	G	A								x		x	x	x	intergenic	-	-
4291518	C	G								x		x	x	x	YM3MPS_38760		Gly101Arg
4323162	C	T									x				YM3MPS_39080		synonymous
4346229	T	C					x								YM3MPS_39290		Cys115Arg
4358238	A	G								x	x	x	x	x	YM3MPS_39400	*irtA*	Ser436Pro
4366236	A	C								x		x	x	x	YM3MPS_39480		Glu63Ala
4439552	G	A									x				YM3MPS_40010	*ppsD*	synonymous
4474175	A	G								x	x	x	x	x	YM3MPS_40170	*fadD29*	synonymous
4584736	A	G								x					YM3MPS_41180	*hycE*	synonymous
4590772	A	G								x	x	x	x	x	YM3MPS_41230		synonymous
4605249	C	T								x	x	x	x	x	intergenic	-	-
4624184	C	G								x		x	x	x	YM3MPS_41550		Ala14Pro
4629482	A	G								x	x	x	x	x	intergenic	-	-
4635428	T	C											x		YM3MPS_41650		synonymous
4649337	C	A								x					YM3MPS_41800		Tyr143*
4659582	T	C									x				YM3MPS_41900		Ile51Thr

4669613	G	A									x	x	x	x	x	YM3MPS_41950		Val188Ile
4671984	G	A										x				YM3MPS_41980		synonymous
4679279	T	C										x				intergenic	-	-
4681323	A	C										x				YM3MPS_42070		Asp68Ala
4727759	T	C									x		x	x	x	YM3MPS_42530		Thr184Ala
4746795	C	G									x	x	x	x	x	intergenic	-	-
4751963	C	T										x				intergenic	-	-
4753976	A	G									x	x	x	x	x	YM3MPS_42790	*ppe51*	synonymous
4775186	G	A										x				YM3MPS_43000		Ala72Thr
4809388	T	G									x	x	x	x	x	YM3MPS_43340		Ile342Leu
4830816	A	G									x		x	x	x	YM3MPS_43480		Val153Ala
4859521	G	A									x		x	x	x	YM3MPS_43800		Asp102Asn
4908374	C	G									x	x	x	x	x	YM3MPS_44290		Ala280Gly
4932814	A	G												x		YM3MPS_44500		Leu490Pro
4949843	T	G									x	x	x	x	x	YM3MPS_44650		synonymous
5040009	G	A									x	x	x	x	x	YM3MPS_45550		synonymous
5088338	G	A									x		x	x	x	YM3MPS_46050	*mprB*	Ser394Phe
5096434	A	G									x	x	x	x	x	YM3MPS_46120	*accA2*	synonymous
5123077	G	A									x	x	x	x	x	YM3MPS_46340		Ala30Thr
5128069	A	G									x	x	x	x	x	YM3MPS_46390		synonymous
5130638	G	T	x													YM3MPS_46400		synonymous
5137349	C	T										x				YM3MPS_46470		Ala136Thr
5137352	G	A										x				YM3MPS_46470		synonymous
5149729	A	G		x												YM3MPS_46610<br>YM3MPS_46620	*pstC2*<br>*pstA1*	*pstC2* : *325Trp<br>*pstA1* : Thr2Ala
5161327	G	A										x				YM3MPS_46700		synonymous
5186276	G	C		x			x			x						intergenic	-	-
5189651	G	A										x				YM3MPS_47030		His260Tyr
5249583	C	T									x		x	x	x	YM3MPS_47580		Leu252Phe
5268320	T	C									x		x	x	x	YM3MPS_47770		Tyr402His
5272378	G	C									x		x	x	x	intergenic	-	-
5283150	G	C						x								YM3MPS_47960		Ser250Trp
5310602	T	C									x		x	x	x	YM3MPS_48290		synonymous
5337327	G	A										x				YM3MPS_48550		synonymous
5355808	G	C									x		x	x	x	YM3MPS_48760		synonymous
5359304	G	A									x		x	x	x	YM3MPS_48790		synonymous
5449719	G	A									x		x	x	x	intergenic	-	-
5480107	G	A									x		x	x	x	YM3MPS_50020		Ser19Asn
5486071	A	C										x				YM3MPS_50090	*emrB*_5	Ile172Leu
5512484	G	A													x	YM3MPS_50310		synonymous
5525731	C	T	x	x	x	x	x	x	x	x	x	x	x	x	x	YM3MPS_50480	*phoR*	Gly422Asp
5526337	A	G													x	YM3MPS_50480	*phoR*	Leu220Pro
5526415	G	A									x					YM3MPS_50480	*phoR*	Ala194Val
5526964	A	G					x									YM3MPS_50480	*phoR*	Leu11Pro
5565675	T	C									x	x	x	x	x	intergenic	-	-
5574342	G	A									x		x	x	x	YM3MPS_50900	*mce4F*	Pro114Ser
5580283	C	T									x		x	x	x	YM3MPS_50950	*mce4A*	synonymous
5594486	G	A											x	x	x	YM3MPS_51080		Pro151Leu
5624344	G	A									x		x	x	x	YM3MPS_51350		synonymous
5641637	T	C									x	x	x	x	x	YM3MPS_51520		Trp348Arg
5643722	C	T									x		x	x	x	intergenic	-	-
5661785	G	A										x				YM3MPS_51720		synonymous
5767491	T	G									x	x	x	x	x	YM3MPS_52680		synonymous
5778249	G	A										x				YM3MPS_52800		Ala757Thr
5812873	G	A									x	x	x	x	x	YM3MPS_53180	*esxA*_1	synonymous
5813598	A	G										x				intergenic	-	-
5815630	G	T			x	x			x							YM3MPS_53220		Arg100Leu
5830162	G	A									x		x	x	x	YM3MPS_53410		Pro32Leu
5871235	C	T						x								YM3MPS_53760		Ala830Val
5874357	A	C									x		x	x	x	YM3MPS_53800		synonymous
5874674	G	C													x	YM3MPS_53800		Gly569Ala
5949080	G	A										x				YM3MPS_54410	*espR*	Glu119Lys
5960203	A	G									x	x	x	x	x	YM3MPS_54550	*gltD*	synonymous
6010476	T	C	x													YM3MPS_54970	*eccE1*	synonymous
6016728	C	T										x				YM3MPS_55010		Ala440Val
6048428	C	G						x								YM3MPS_55300	*yidC*	synonymous
